# Gaps and Doubts in Search to Recognize Glioblastoma Cellular Origin and Tumor Initiating Cells

**DOI:** 10.1155/2020/6783627

**Published:** 2020-07-22

**Authors:** Aneta Wlodarczyk, Dagmara Grot, Ewelina Stoczynska-Fidelus, Piotr Rieske

**Affiliations:** Department of Tumor Biology, Medical University of Lodz, Zeligowskiego 7/9, 90-752 Lodz, Poland

## Abstract

Cellular origin of glioblastoma (GB) is constantly discussed and remains a controversial subject. Unfortunately, neurobiologists are not consistent in defining neural stem cells (NSC) complicating this issue even further. Nevertheless, some suggestions referring to GB origin can be proposed based on comparing GB to central nervous system (CNS) cells. Firstly, GB cells show *in vitro* differentiation pattern similar to GFAP positive neural cells, rather than classical (GFAP negative) NSC. GB cells in primary cultures become senescent *in vitro*, similar to GFAP positive neural progenitors, whereas classical NSC proliferate *in vitro* infinitely. Classical NSC apoptosis triggered by introduction of IDH1R132H undermines hypothesis stating that IDH-mutant (secondary) GB origins from these NSC. Analysis of biological role of typical IDH-wildtype (primary) GB oncogene such as EGFRvIII also favors GFAP positive cells rather than classical NSC as source of GB. Single-cell NGS and single-cell transcriptomics also suggest that GFAP positive cells are GB origin. Considering the above-mentioned and other discussed in articles data, we suggest that GFAP positive cells (astrocytes, radial glia, or GFAP positive neural progenitors) are more likely to be source of GB than classical GFAP negative NSC, and further *in vitro* assays should be focused on these cells. It is highly possible that several populations of tumor initiating cells (TIC) exist within GB, adjusting their phenotype and even genotype to various environmental conditions including applied therapy and periodically going through different TIC states as well as non-TIC state. This adjustment is driven by changes in number and types of amplicons. The existence of various populations of TIC would enable creating neoplastic foci in different environments and increase tumor aggressiveness.

## 1. The Cellular Origins of GB

According to WHO Classification of Tumors of the Central Nervous System (CNS) from 2007, glioblastomas (GB) were divided into primary and secondary subtypes. Revision made in 2016 modified the classification, distinguishing GB subtypes based on the IDH genes mutation status [1]. As stated in new guidelines, primary GB was replaced by IDH-wildtype GB, whereas secondary GB by IDH-mutant GB. However, due to the review character of this article and referring to archival data prior to 2016 report, the previous nomenclature (primary and secondary GB) will also be used.

Establishing the origin of GB cells is essential not only for basic science purposes but also to develop better therapies [2]. The first difficulty in determining the origin of GB cells lies in the lack of an unambiguous defining of what neural stem cells are and what they are not. How important it is to define these entities shows an article written by Bhaduri et al. [3]. Authors suggest that GB originates from radial glial cells, more specifically, outer radial glial cells (oRG). However, there is a dispute whether radial glial cells are stem cells or progenitors. At least *in vitro* radial glial cells usually do not meet the criteria of stem cell definition because their proliferation potential is very limited. Pollard et al. indicated that radial glial cell lines derived from pluripotent stem cells were immortal; however, in other articles radial glial cells were recognized as cells with limited *in vitro* and even *in vivo* proliferation potential [4–7]. Unfortunately, there are no commercially available (not genetically engineered) immortal human GFAP positive cell lines. At the same time, it is easy to get access to immortal classical GFAP negative neural stem cells. Since GFAP negative neural stem cells (NSC) were historically specified first, these cells were referred here as classical NSC. These NSC can proliferate in cell culture conditions infinitely [8–12]. On the other hand, *in vitro* division limits do not necessarily mean that radial glia are not stem cells. One would suggest that we are not able to culture these cells properly *in vitro* and hiding their ability to self-renew in these conditions (Table 1). However, developmental biology analyses suggest that this is a more complicated issue. Probably the loss of division capacity shown by radial glial cells *in vitro* has something to do with radial glia transition to astrocytes observed during final stages of CNS development [40]. Although radial glial cells differentiation into neurons depends on asymmetrical divisions with self-renewal [6, 19], their differentiation or transition to astrocytes is not divisions dependent [20]. Simply, after the CNS development, many radial glial cells turn into astrocytes [40, 41]. This shows that radial glial cells do not fulfill criteria of typical stem cell.

Since radial glial cells become astrocytes after development, the next question arises. Does radial glia exist at all in the normal CNS of adults? Bhaduri et al. together with other groups state that there is no radial glia in the normal CNS of an adult [3, 21–24]. Then how come GB in adults can be originated from radial glial cells? Bhaduri et al. firstly proposed that “developmental programs are reactivated in the tumors” [3]. Next, they suggested, “While radial glia are not believed to be present in the normal adult human brain it is possible that there is a latent or quiescent population that can give rise to GSCs and glioblastoma or that a neuronal or glial cell de-differentiates into a oRG-like cell to initiate tumors” [3]. This last statement leaves the reader with some ambiguity. If the process of dedifferentiation is required, then how is it induced? And is radial glial cell indeed the cell from which GB originates? Maybe there is another cell which dedifferentiates during first stage of glioblastomagenesis to radial glia-like cells as a result of mutation. Interestingly, Ghashghaei et al. showed that high expression of ErbB2 in astrocytes enables them to regain radial glial features [37]. This shows that results of different studies indicating that GB is derived from astrocytes and radial glia can be coherent not contradictory.

Another recently published article by Lee et al. with very elegant single-cell NGS study proves that primary GB (IDH-wildtype) arises from subventricular zone (SVZ) astrocyte like NSC [25]. The question here is whether the cells described by Lee et al. are the same cells that Bhaduri et al. characterized on the basis of single-cell transcriptomics. Intuitively, this seems quite inconsistent because human oRG, as the name suggests, are located in the outer subventricular zone (OSVZ) [42]. However, Pollen et al. as well as Reillo et al. suggested that the ventricular zone (VZ) and adjacent inner SVZ contain mixed populations of ventricular radial glial cells (vRG) and oRG cells destined to migrate to the OSVZ [43, 44].

**Figure 1:**
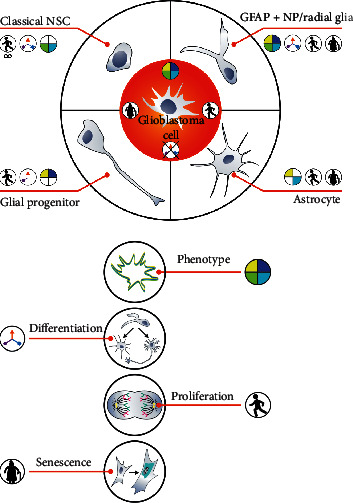
Similarities and differences between glioblastoma cells versus astrocytes, glial progenitors, GFAP + NP (radial glia), and classical NSC. GB cells resemble GFAP + NP the most in terms of phenotype and susceptibility to senescence. Classical NSC and astrocytes do not show the expression of GFAP and SOX2, respectively. Although GFAP + NP radial glia and GB show similar differentiation features, GB differentiation is blocked. Astrocytes and glial progenitors differentiate in the same way as GB cells. Classical NSC, unlike GB cells, proliferate *in vitro* far beyond the limit. GFAP + NP and GB cells quickly become senescent *in vitro*.

**Figure 2:**
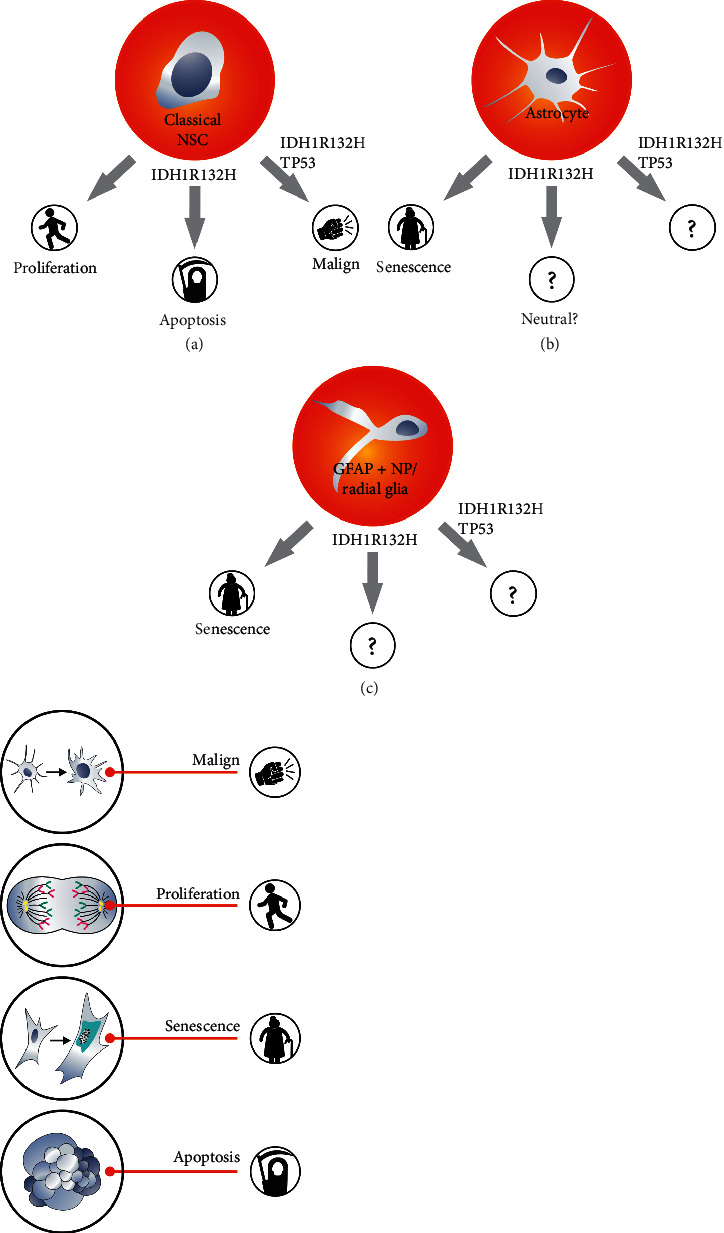
Explaining the origin of secondary glioblastoma requires IDH1R132H analysis; however, this oncogene influence on cells other than NSC was not examined profoundly.

*In vitro* studies in general could be helpful in testing above listed cells as putative origins of GB due to the possibility of using such techniques as CRISPR to mimic tumorigenesis. Unfortunately, classical NSC (as nestin and SOX2 positive and GFAP negative cells) are the most commonly studied in these conditions, due to the simplicity of their *in vitro* culturing methods compared to astrocytes, radial glia, or GFAP positive neural progenitors (NP) culturing methods (Figure 1) [13, 28, 29]. Classical NSC *in vitro* adjustment comes from the above-mentioned self-renewal ability [8]. Contrary to GFAP negative neural stem cells, GFAP + NP (or, probably, GFAP + NSC) and glial progenitors do not have that type of ability to self-renew and quickly become senescent under *in vitro* conditions (Figures 1 and 2) [14, 15, 45]. Astrocytes also undergo senescence *in vitro* [38]. Radial glial cells have not been tested directly for senescence yet, but these cells can transform into astrocytes. Lack of easier *in vitro* cell models to be analyzed other than classical NSC leaves many gaps in *in vitro* testing hypotheses about the origin of GB from nonclassical NSC or GFAP positive progenitors and astrocytes (Figure 2).

Researches convinced that radial glia or GFAP positive cells fulfill neural stem cells definition, may ask if GB originate from GFAP positive NSC or GFAP negative NSC [22], and such work as Lee et al. based on single-cell NGS will make them focus on GFAP positive cells, at least in the field of the primary (IDH-wildtype) GB [25].

### 1.1. Are There Two Origins of GB?

When considering the cellular origin of GB, the differences between secondary and primary tumors should be realized. The secondary GB develops from grade I astrocytoma through grades II and III astrocytomas. The primary GB in return does not develop from low-grade tumors [46, 47]. Therefore, different cellular origin of these two types of GB seems to be possible (Table 1). There is a growing evidence that, although histologically similar, GB with and without IDH1 mutation appear to represent distinct disease entities that arise from separate cell types of origin at least as a result of largely nonoverlapping sets of molecular events [48, 49]. Moreover, the observation of these tumors location suggests that oligodendrogliomas, astrocytomas, and subsequent secondary glioblastomas originate from precursor cells located in or migrating to the frontal lobe [50–52]. Barami et al. performed a retrospective radiographic analysis of 100 patients with gliomas. According to MRI scans, they demonstrate that in approximately 93% of cases, indicated lesions were contacted at least with one region of the SVZ, independent of the glioma size or mass effect, thereby highlighting a correlation between GB and the subventricular zone [53]. Similarly, the different clinical outcome as well as different age groups indicate the different origin of these two subtypes of GB [54]. Interestingly, not only primary and secondary GB can be of a different origin. Verhaak et al., who performed an integrated genomic analysis of GB specimens and characterized four different GB subtypes (proneural, neural, classical, and mesenchymal) suggested one of the possibilities is that tumors in specific subtypes develop as the result of different cells of origin [55]. This general idea was further supported by Alcantara et al., however, with two phenotypically and molecularly distinct main GB subtypes [56]. On the other hand, it is possible that there is a common cell of origin, such as GFAP + NP/radial glia or classical NSC, and the classes of GB arise from distinct differentiation paths. It should be however noted that around 8% of GB samples score for more than one subtype [57].

The hypotheses considering the origin of GB are very difficult to be definitely verified thoroughly because of the inability to real-time tracking of this process in humans. Nevertheless, some studies shedding light on this aspect have been published [46, 47, 58]. These included, for example, mutation pathway from low-grade astrocytoma to secondary glioblastoma. Unfortunately, these results did not include the first stage of the process (the formation of grade I astrocytomas).

When secondary (but not only) GB are considered especially, progress in cell reprogramming technology (obtaining induced pluripotent stem cells (iPSC) from mature cells) puts the problem of GB development in a different context [59]. Epigenetic reprogramming during the development of secondary GB seems likely when considering the effects of various oncogenes, for example, IDH1R132H. Importantly, IDH1R132H is observed only in secondary GB [60] and leads to epigenetic changes [61, 62]. This supports the hypothesis that there are at least two origins of glioblastoma, different for secondary and primary GB. In secondary GB, the possibility of astrocytes reprogramming deserves more attention. Some authors suggest that generation of glioma is a result of “neonatal astrocytes” transformation [39, 63]. However, others, for example, Modrek et al., indicated that even low-grade astrocytomas arise from classical (GFAP negative) NSC) [64]. Surprisingly Modrek et al. admitted that the IDH1R132H mutation has proapoptotic activity in these NSC. Similar observations were made by other researchers [30, 31]. Apparently, alterations subsequent to IDH1, including the loss of TP53, reverses effects of IDH1R132H mutation, and both mutations show positive impact on the survival of mutated classical NSC [64]. It shows that alterations subsequent to IDH1R132H change its influence from negative (antisurvival) to positive (prosurvival) but Kleiheus et al. proved that secondary glioblastoma tumorigenesis takes years [65]. Since IDH1R132H alone promotes apoptosis in classical NSC, then arising question is how NSC carrying this mutation survive, until additional mutations occur. Only few researches examined IDH1R132H influence on astrocytes and suggested neutral impact of this gene on these cells (Figure 2) [66]. We do not know works showing IDH1R132H influence on radial glia, GFAP + NP, or GFAP + NSC.

### 1.2. What Should We Know from Animal Studies? Are Results from Animal Studies Convincing?

Additional data for further analyses of the origin of GB come from genetically modified (engineered) animal studies. It is known that rodent tumors are significantly different from human neoplasia. Therefore, this model is not entirely convincing. Here, the question of whether NSC and neurogenesis occur in adult human brain arises. Sorrells et al. demonstrated that hippocampus during adulthood do not generate new neurons, in opposition to examined rodents, whereas hippocampal neurogenesis still occurs during life [22]. Moreover, the other histopathological results performed by Sanai et al. exhibited that migration of neural immature progenitors by SVZ restoral migratory system (RMS) to the olfactory bulb (OB) has been disappearing between the 6th and 18th month of life, thereby for a very long time before the usual diagnosis of glioblastoma [67]. These findings question the appropriation of mouse NSC-derived model results transfer to humans. However, Boldrini et al. performed a whole-autopsy hippocampus from healthy different-aged (14–79) humans [68]. They demonstrated that intermediate neural progenitors and immature neurons in dentate gyrus were still detected even in adult person; however, their numbers were inversely proportional to the age [68]. It is very difficult, and often impossible, to conduct more complex studies on humans, which make the animal models as a basis of our knowledge in this field with an obvious reservation that obtained results will be not always relevant in human case.

In general, genetically modified animals' studies suggest that many types of murine CNS cells can represent the origin of GB. Based on these studies GB can originate from either astrocytes, oligodendrocyte precursor cell, neural progenitors, or neural stem cells. Bachoo et al. demonstrated that combined loss of p16INK4a and p19ARF triggers dedifferentiation of astrocyte in response to EGFR activation, and that together leads to gliomagenesis [39]. Singh et al. used the same model to show that oncogenes trigger transcriptional regulatory circuit. They propose that glioblastomas are resistant to EGFR tyrosine kinase inhibitors (TKI) because of this circuit further autonomy [69]. Alcantara et al. used nestin positive NSC/progenitors and transient silencing of TP53, NF1, and PTEN in these cells using the tamoxifen-induced Nestin-Cre system resulting in glioma formation [16]. However, the silencing of the same genes in the nonneurogenic zone did not lead to tumorigenesis [16]. Similarly, deletion of TP53, Pten, and/or Rb in NSC from SVZ, but not in peripheral astrocytes, promoted mouse gliomas development [32]. In another case, a model of NOD/SCID mice with implanted PTEN-null human NSC line, obtained with utilization transcription activator-like effector nuclease- (TALEN-) mediated homologous recombination (HR), and upregulation of PAX7 was used. In this model PAX7 promoted NSC transformation and corresponded to malignancies of developed GB [33]. Alternatively, Hongwu Zheng et al. demonstrated that TP53, PTEN, and EGFR mutations found in SVZ NSC led to the development of glioblastoma-like tumors in mice [70]. Holland et al. showed that combined activation of Ras and Akt in neural progenitors induces glioblastoma formation in mice [17].

Referring to secondary glioblastoma, Philip et al. delivered IDH1R132H to nestin-expressing cells using RCAS/TVA glioma model. IDH1R132H promoted transformation of nestin-expressing cells exposed to PDGFA and showing loss of CDKN2a, ATRC, and PTEN [66]. Bardella et al. suggested that IDH1R132H conditional, inducible expression in the adult mouse SVZ stem cell niche causes cellular and molecular features associated with brain tumorigenesis [71]. Those animal experiments data support conception of GB origin from many types of cells.

## 2. Phenotypical Similarities between GB Cells, Neural Stem Cells, and Neural Progenitors

**Figure 3:**
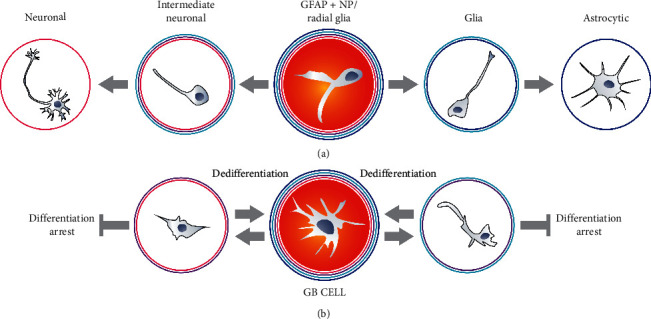
GFAP + NP cells differentiate similarly to glioblastoma cells. However, GB cell differentiation appears to be blocked at the early stages. GFAP + NP radial glial cells also differentiate in a very distinctive way due to the presence of GFAP. The loss of GFAP is essential for NP to differentiate into neurons. GB cells exhibit similar differentiation characteristics but appear to be blocked at the early stages. GB cells, in contrast to NSC or GFAP + NP, can differentiate and dedifferentiate.

GB phenotype markers recognized by pathologists (excluding IDH1R132H) are identical for secondary and primary GB. Today, existence of these two types of GB tumors is obvious. But before the IDH1R132H discoveries, it was questioned, because of their phenotypic identity [39]. So, this paragraph applies to both IDH-wildtype and IDH-mutant glioblastomas. Phenotypical similarity between any GB cells and NSC can be considered as argument supporting NSC origin of GB. Such similarities undoubtedly exist [72]. However, we can ask whether GB cells actually resemble classical NSC or neural progenitors/radial glia, and so on, that share some of the features with classical NSC. Indeed, GB cells coexpress SOX2 and Nestin but also express GFAP [73]. This is a typical phenotype for GFAP positive neural progenitors or radial glia and not for classical NSC or glial progenitors [3, 74]. Considering differentiation derivatives, GFAP positive neural progenitor (GFAP + NP) cells or radial glia may play a role in the formation of both, secondary and primary GB, the same as the case of classical NSC (Figure 1). Importantly, GFAP + NP or some types of radial glia are source of neurons, astrocytes, and oligodendrocytes, that is, the same cells that are derived from typical well-established *in vitro* NSC [18, 34–36]. However, GFAP positive cells differentiate in a very characteristic way due to the presence of GFAP. In the case of radial glial cells changing to astrocytes, some authors propose even term transformation to astrocytes since GFAP was simply preserved in derivative cells [26, 27]. The loss of GFAP is essential for cell such as NP or radial glia to differentiate into neurons or oligodendrocytes [13, 45]. GB cells show similar differentiation pattern to radial glia or in general GFAP positive progenitors and not to classical NSC. GB cells differentiation process appears to be blocked at the early stages (Figure 3) [73, 75, 76].

Moreover, Bhaduri et al. show that glioblastoma derived primary cells undergo mitotic somal translocation, a process previously observed only during human development [3]. It suggests reactivation of developmental programs characteristic for radial glial cells in glioblastoma cells. It is another phenotypical similarity between GB cells and radial glial cells [35, 77, 78].

Unlike classical neural stem cells, GFAP + NP, radial glia, and glial progenitors do not have the capacity to self-renew *in vitro* as GFAP negative NSC [5, 7, 14, 15, 29, 45] and quickly become senescent in those conditions [79]. Importantly, when considering the susceptibility to senescence, GB cells resemble GFAP positive neural progenitors rather than GFAP negative classical NSC (Figure 1) [29, 79–81].

The above described phenotypical similarities between GB cells and GFAP positive cells can be important, where therapy for GB patients is designed. A particular type of mitosis and even more susceptibility to senescence may appear to be the Achilles' heel of GB cells.

It turns out that classical GFAP negative NSC exhibit much higher proliferative potential than GB cells *in vitro*. The process of GB cells *in vitro* senescence applies to both secondary and primary GB [80]. In general, even pericytes or the so-called glioma associated stromal cells (GASC) are able to proliferate longer than GB primary cells *in vitro* [80]. In the majority of GB, there are no senescence-resistant cells, or cells able to establish stable cell line (dividing infinitely). Although the mechanism of senescence is not fully explained, at least part of the senescence phenomenon is a consequence of mitotic catastrophes [82]. Genetic modifications of GB cells leading to the expression of TERT or SV40 do not prevent the senescence [82]. Stable cell lines (proliferating for years) can therefore be established from minority of GB. GB cell lines with endogenous IDH1R132H mutation, as well as EGFRvIII-positive cell lines are extremely rare. Consequently, researchers have to use such models as intracerebral PDX [83, 84].

The facts that GB cells do not behave *in vitro* like immortal classical NSC, and especially that pericytes proliferate longer than GB cells, are very intriguing. These observations show that our perception of the development of GB is still very superficial. *In vitro* observations and phenotypical similarities between GB cells and cells such as radial glia suggest that GFAP negative NSC are not the most logical target for carcinogens. Naturally, *in vivo* and *in vitro* conditions are completely different. However, *in vitro* observations cannot be ignored. Based on commonly accepted models of tumorigenesis or models of the NSC-based origin of GB, it is difficult to explain why NSC can proliferate freely *in vitro* whereas GB cells rapidly undergo senescence. There are no senescence-resistant GB cells *in vitro* (except for a minority of cases where stable cell lines can be obtained).

Data presented here suggests that GB is derived from GFAP positive progenitors or GFAP positive NSC rather than from GFAP negative classical NSC.

## 3. The Biological Role of Oncogenes and the Supposed Origin of GB

**Figure 4:**
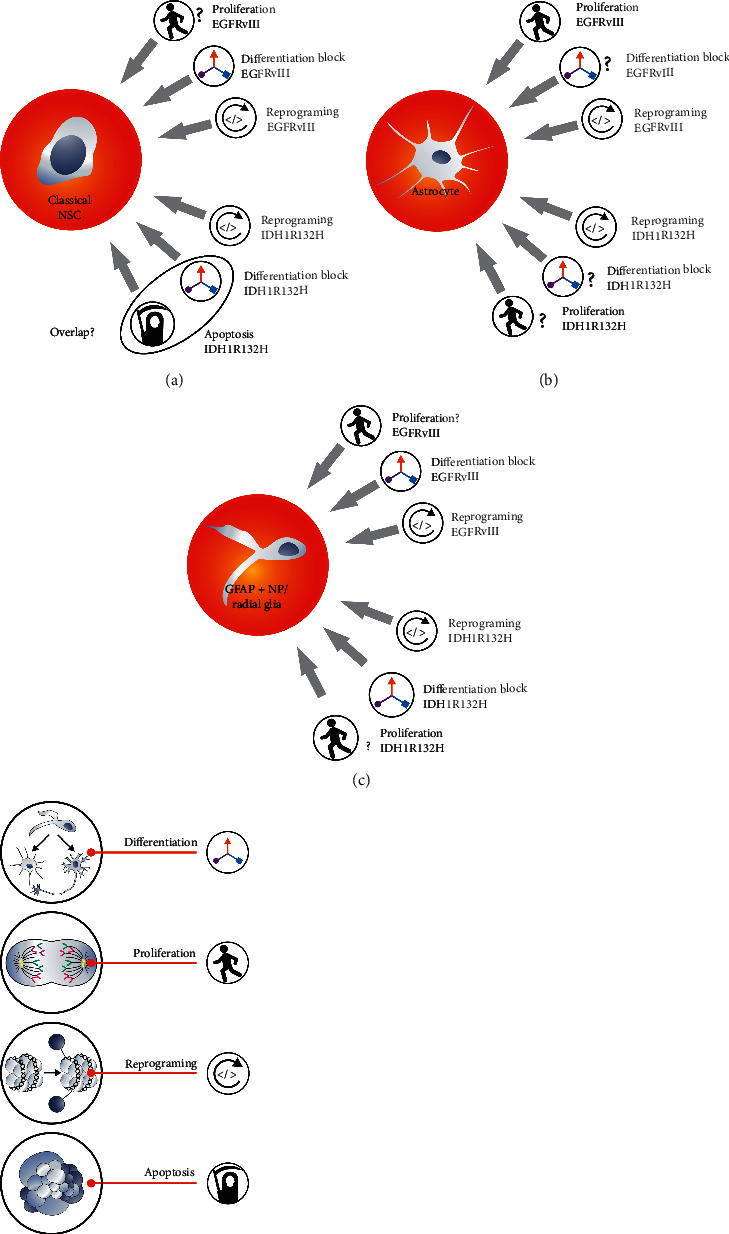
Actions of two selected and mutually exclusive oncogenes EGFRvIII and IDH1R132H suggest origin of primary and secondary glioblastoma, respectively.

This paragraph refers to two oncogenes: EGFRvIII more characteristic for primary GB (IDH-wildtype) [55, 85–89] and IDH1R132H for secondary GB [1, 55, 60, 90, 91]. Oncogenes such as EGFR mutants are considered to act as accelerators of proliferation and inducers of immortalization. However, classical GFAP negative NSC divide far beyond the Hayflick's limit *in vitro*; NSC are immortal [8]. Unlimited proliferative potential of these NSC and *in vitro* senescence of GB cells allow reasking what the oncogene mechanisms of action in GB are and whether GFAP negative NSC are the target for carcinogens. It seems that if these neural stem cells (such as these used in *in vitro* cultures [8]) were to be the target for carcinogens during gliomagenesis, the feature of excessive proliferation may be observed before oncogenes mutation, in other words, why the cells proliferation should be dependent on oncogenes, if classical NSC proliferation rate is originally very fast (Figure 4). Discussion on the role of oncogenes is also complicated due to the fact that *in vitro* senescence of GB cells resembles senescence of GFAP + NP [80, 81]. In addition, several studies including our own research suggest a high importance of a block of differentiation in GB (Figure 3) [73, 75, 76, 92]. In case of GFAP + NP or radial glia, inhibition of differentiation makes more sense than in case of astrocytes (Figure 4). This differentiation block may be oncogene-dependent [76, 92, 93]. Therefore, GB cells differentiation inhibition seems to be more important than increased proliferation rate (Figure 4). Alternatively, general opinion about pro-proliferative role of oncogenes is correct; however, classical NSC are not the origin of glioblastoma. If cells such as astrocytes, GFAP + NP, radial glia, and GFAP + NSC are the origin of glioblastomas, then pro-proliferative role of oncogenes such as EGFRvIII appears to be more rational at least based on *in vitro* observations. Bhaduri et al. and others suggested that radial glia proliferation activity can be restored at the early stages of glioblastomagenesis which again fits better to the role of EGFRvIII in these cells than in classical NSC [3, 35, 94, 95]. In accordance with that, Ghashghaei et al. showed that ErbB2 enables astrocytes to regain radial glial features [37].

Obviously, not only proliferation rate is crucial for tumor cells. The proliferation of normal cells including classical NSC is strictly controlled and can be inhibited by environmental factors [96], whereas tumor cells proliferate more autonomously [69, 97]. It means that constitutively active EGFRvIII can promote general biological autonomy of neoplastic cells, independently of their origin [98, 99]. Anyway, GB developed from classical NSC are expected to be more aggressive than those developed from radial glial cells. Uncontrolled proliferation of classical NSC should lead to extremely rapid tumor growth [16, 32, 100, 101].

IDH1R132H is another important glioblastoma oncogene. IDH1 mutation is proposed to be the first mutation that occurs during the formation of secondary GB that affects all, or almost all, secondary GB [91]. Tumors such as astrocytomas, from which secondary GB originate, grow relatively slowly [46, 47]. It takes many years for astrocytoma to turn into glioblastoma [46]. Therefore, the primary role of IDH1R132H is not to increase proliferation rate (Figure 4). The effect of the IDH1R132H mutation on cell reprogramming is important (Figure 4) [58, 64, 102]. How does analysis of IDH1R132H function address the issue of GB origin? Researches of Modrek et al. and Ying Zhang et al. as well as our own data indicate that introduction of IDH1R132H into classical neural stem cells can cause quite complex effects. It can even trigger apoptosis [30, 31, 64]. Moreover, it is unlikely for cells proliferating as slowly as grade-one astrocytoma cells do to be derived from classical NSC showing high proliferative capacity. Thus, it is assumed that IDH1R132H mutations may affect cells other than classical NSC to initiate gliomagenesis. Moreover, it can suggest that primary and secondary GB may have different origin. Lu et al. proposed that IDH1R132H blocks differentiation [103]. This type of mutant mechanism of action seems to be more relevant in NSC or GFAP + NP than in astrocytes; however, Rosiak et al. showed that differentiation blockade may be misinterpreted with proapoptotic activity of IDH1R132H, or both actions occur simultaneously in classical NSC overexpressing this oncogene (Figure 4) [31].

To sum up, activation of typical for primary GB oncogene activation (EGFRvIII) makes more sense in cells other than classical NSC; however, this oncogene helps any cell to become more autonomous. Proapoptotic activity of IDH1R132H in classical NSC suggest that other cells such as astrocytes, GFAP positive progenitors, or radial glia should be considered more carefully as potential origin of secondary GB.

## 4. Are Tumor Stem Cells or Tumor Initiating Cells Marginal Population of GB?

The next question is whether GB contains tumor stem cells (TSC) or tumor initiating cells (TIC). The TSC/TIC theory evolves. Lately, Yang et al. proposed a term, tumor survival cells [104]. Capp et al. suggested that cancer stem cell refers rather to status than entity [105]. Bhaduri et al. used even expression profiling of stem cells population suggesting that group of certain cells is required to rebuild this tumor [3]. One of the potential meanings is that TIC are the cells that induce tumor formation at the initial stages of its development, before all mutations have occurred [106]. Another definition says that it is the population of cells within a fully formed tumor that allow its regeneration or create new neoplastic foci [107]. This chapter focuses on the second meaning: tumor-derived stem cells enabling formation of the whole tumor after being introduced into normal nervous tissue or even to other tissues. Naturally, the potential NSC origin of GB cannot determine whether GB contains stem cells or tumor initiating cells. One can imagine that GB does not consist of TSC even though it is NSC-derived, or, on the other hand, it may be assumed that although GB is derived from astrocytes, it contains TSC subpopulation. Firstly, to address this issue, GB cells can be compared to classical NSC or progenitors. It was suggested that GB cells resemble GFAP positive neural progenitors [64, 73]. In some cases, even more than 50% of GB cells with markers of GFAP positive neural stem cells or glial progenitors was directly isolated from tumors [73]. It was also demonstrated that these GFAP positive/SOX2 positive cells express IDH1R132H [81]. The similarity to GFAP + NP does not mean that the majority of GB cells are TIC (TSC), but it is worth noting. The idea of a cancer stem cell (tumor stem cells) seems to be contradicted by the commonness of such cells in the tumor. The TSC population is usually described as a marginal (side population) [108]. The tumor is compared to normal tissue in which stem cells represent only a small percentage of cells and there is a hierarchy of cells based on the level of differentiation: stem cells, progenitors with increasingly lower differentiation capacity, and, finally, mature cells. However, normal differentiation is in general an irreversible process (iPSC generation is a biotechnological process), whereas cyclic change of phenotypes in tumor cells, similar to cyclic differentiation and dedifferentiation, is very likely [109–111]. Inhibition of advanced GB differentiation makes this cyclic reversion even more plausible (Figure 3). It means that TIC phenotype can be sometimes observed only inside population of cells, but the majority of GB cells can switch sooner or later into the TIC (Figure 2). There is no obvious answer to the question why we should stick to the idea of tumor as a “tissue,” in which only a small part of cells may have the ability to generate or regenerate the tumor. Glioblastoma, unlike tissues, does not require various types of mature cells (irreversibly differentiated cells), to perform very complicated tasks. This does not mean that different tumor cells do not perform different tasks at all, but it seems pointless to compare their specialization to the tissue specialization of, for example, oligodendrocytes, astrocytes, and neurons. To this end, the term tumor initiating cell seems to make more sense than tumor stem cells, unless data suggesting that vessel-like structures in GB are formed from glioblastoma cells will be confirmed [112]. This process is called vascular mimicry, and GB cells differentiating vessels cells could be considered as fulfilling better tumor stem cells definition. The formation of blood vessels is undoubtedly a specialization of a very high degree. However, it seems that the vascular mimicry dispute will continue for quite some time [113].

**Figure 5:**
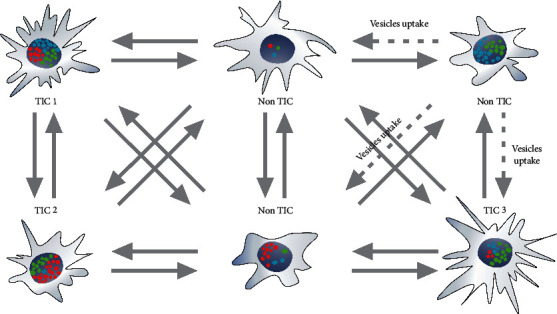
Phenotypic heterogeneity of glioblastoma may result from genotypic heterogeneity associated with a different number of extrachromosomal amplicons and their different composition. Various types of tumor initiating cells can convert/transit from one type to another or to non-TIC cells, whereas non-TIC cells can convert/transit to TIC due to the changes in types of amplicons as well as their numbers and epigenetic changes. The presence of different TIC increases tumor aggressiveness, since neoplastic cells are able to invade different environments and survive many environmental changes including applied therapy. Amplicons can be transported in extracellular vesicles. Separated amplicons contain genes such as EGFR and EGFRvIII, MDM2, PDGFR, or c-MYC.

The presence of TIC side population in GB can be also debated from genetic and epigenetic point of view. Genes mutated in all GB cells in tumor such as PTEN, IDH1R132H, and even TP53 are supposed to sustain cells in stem cell status [114–118]. Since all tumor cells have mutations that promote stemness, then why only a small part of the tumor may possess the features of a stem cell [119–122]? In actual fact, there are significant exceptions from the genetic homogeneity of tumor cells in GB, coherent with differentiation hypothesis: cyclic transition from TIC to non-TIC state. Many genes undergo extrachromosomal amplification, and their amplification is considered as a driver and a cause of GB heterogeneity [123, 124]. EGFRvIII is an example of such amplified gene [125]. The expression of this oncogene, which may be involved in stem-like features [92], usually does not occur in all GB cells, nor does massive extrachromosomal amplification [125]. Extrachromosomal amplicons are structures that challenge the concept of genotype stability in tumor cell. In an individual cell, there are from a few to several hundreds of specific amplicons. Furthermore, a single amplicon may contain from several to several hundred copies of the gene [123]. Next, the cells may have different sets of active and massively amplified genes within extrachromosomal amplicons. This results in a large number of different combinations of amplicon genotypes within a tumor (Figure 5) [124]. Therefore, discussions on a single genotype favoring stem-like status in the tumor are a simplification, especially in the case of such tumor as GB where oncogene amplification is a fairly common phenomenon. Our data indicate that there are cases of GB in which only a small percentage of cells show amplified EGFRvIII [126]. Different researchers draw different conclusions from this fact. Some suggest that EGFRvIII is a marker of GB stem cells [92]; others undermine its relevance in the later stages of the tumor development [127]. Our analyses, however, suggest that this gene is important at very advanced stages of glioblastoma [99]. EGFRvIII is obviously not the only gene to be amplified in glioblastoma. Other amplicons, for example, MDM2 along with several genes [128, 129] and c-MYC or PDGFRA amplicons, can also affect tumorigenesis [130]. Heterogeneity caused by the presence of different extrachromosomal amplicons, their different numbers, and epigenetic changes may favor reversible shifts from non-TIC to TIC. Thus, it is possible to propose the way cells can switch/convert between different phenotypes and acquire or recover TIC phenotypes [123, 124]. Interestingly, extracellular vesicles can transfer amplicons from cells with amplicons to cells lacking them (Figure 5) [131]. The genotype of cells with amplicons (especially various extrachromosomal amplicons) is very flexible. In fact, at the genotype level, different GB cells that have at least one amplicon with different oncogenes are capable of rapidly evolving into different TIC. The environmental change influencing amplicons may also be a consequence of the administration of an appropriate antineoplastic therapy [132]. Different therapies can target different cells, which then rebuild the tumor. Still, some scientists have been tempted to design a specific TSC/TIC-targeted therapy [133, 134]. The flexibility of TIC and the cyclical transition from non-TIC to TIC could become ‘a tumor response' to such attempts. Not only are extrachromosomal amplicons characterized by constant changes in number and composition, but also reprogramming suggests that TIC features can periodically appear in the cells. IDH1R132H-dependent reprogramming effects or epigenetic changes are unquestioned. This protein increases the concentration of 2-hydroxyglutarate, which affects the activity of enzymes such as TET2 [62]. Therefore, the transition between the phenotypes can potentially be achieved, due to IDH1R132H triggered epigenome changes or by amplicon-associated genome changes. Both extrachromosomal amplifications and mutations of genes such as IDH1 can enable cyclical transition from the non-TIC to TIC state.

Finally, we should also consider not only switches between non-TIC and TIC, thanks to amplicons and IDH1R132H, but also transitions from TIC1 to the TIC2, TIC3 states, and so on (Figure 5). Initially, the comparison of TIC to NSC or multipotent stem cells seems to be natural but imposes unnecessary restrictions on the TIC, since they do not form tissues. Unlike normal tissue, tumors can benefit from a variety of TIC. The idea of GB or cancer stem cells homogeneity in general comes from the assumption that the hierarchy of tumor cells resembles the hierarchy of cells in normal tissue. However, such a hierarchy does not seem to be helpful in glioblastomas or malignancies in general. In neoplasia, the reconstruction of tumor structure or the formation of metastasis may require cell invasion to and proliferation in various environments as opposed to normal stem cells, which operate effectively in a well-defined niche. TIC in one place may not be able to fulfill such a role elsewhere in the body or generally in more than one environmental condition. It shows that the existence of various populations of TIC able to create neoplastic foci in different conditions increases tumor aggressiveness. Consequently, we can distinguish the population of TIC1, TIC2, and so on, which means the existence of different TIC for different environments, but also narrowing of the TIC population for a given environment at the same time. It is worth realizing that the cells responsible for tumor rebuild are and simultaneously are not “elite.” Recent research confirmed phenotypic heterogeneity of the TSC population [3]. Bhaduri et al. showed glioblastoma stem cells (their term) heterogeneity within and across glioblastomas to detect that they do not consider amplicons but single-cell expression profiling [3].

An additional aspect arising in the context of TSC or TIC is metastasis formation. Some researchers suggest that TSC (TIC) are responsible for metastasis [135], which relate to the tumor formation in the new environment. GB, unlike many other neoplasia, generally does not metastasize, or its metastasis cannot be observed. It is difficult to determine whether this is due to the fact that GB cells are unable to initiate metastasis or to the fact that GB patients die soon before any metastasis can occur [136, 137]. There are only single reports of GB metastases [136, 137]. The lack of these observations deprives the GB TIC discussion of the concept of metastasis.

In conclusion, in order to settle the question of the origin of GB, neural stem cells, radial glia, and neural progenitors should be defined. Moreover, for these considerations, we suppose to distinguish primary and secondary GB origin. Based on published data, we suggest that glioblastomagenesis starts from GFAP positive cells rather than from classical GFAP negative NSC. However, many questions still await their answer, for example, whether radial glial cells are present in adults brains, how come radial glial cells can be the origin of GB if they are absent in CNS, or what would happen after expressing IDH1R132H or EGFRvIII in astrocytes or radial glial cells. Different proliferation capacity of classical NSC versus GFAP positive cells suggests that the origin of GB matters for designed therapies.

Very likely, GB contains TIC cells. There may be several types of GB TIC and their diversity may be related to the variable number of amplicons and their composition.

IDH1R132H triggers apoptosis in NSC. IDH1R123H along with TP53 and ATRX enhances invasiveness based on NSC analyses. Astrocytes and GFAP + NP quickly become senescent *in vitro* (b,c) which makes these cells less willingly examined. NSC proliferate quickly whereas astrocytoma cells do not. Question marks shows tests which were performed occasionally or never on astrocytes and GFAP + NP.

EGFRvIII is able to initiate reprogramming, activate proliferation, and inhibit differentiation. IDH1R132H is able to reprogram cells and to inhibit differentiation. In general, IDH1R132H positive influence on proliferation is not expected. Moreover, IDH1R132H triggers apoptosis of classical NSC. Some data suggest that inhibition of classical NSC differentiation and apoptosis caused by IDH1R132H are overlapping. IDH1R132H- or EGFRvIII-dependent inhibition of differentiation makes more sense if classical NSC or GFAP + NP would be the origin of GB (question mark next to astrocytoma). EGFRvIII-dependent activation of proliferation makes more sense if GB origins from astrocytes or GFAP + NP, since classical NSC shows high proliferation rate (question mark next to NSC).

## Figures and Tables

**Table 1:** Different cell types as origin in GB formation: comparison of selected features.

Different cell types as origin in GB formation						
	Specific markers	Proliferation *in vitro*	Differentiation *in vitro*	Glioma development in genetically modified animals	Other indicators pro and against origin of GB	References

GFAP + NSC/NP	Similar to GB cells: GFAP+ SOX2+ NESTIN+	Limited, similar to GB cells *in vitro*	Multipotent differentiation similar to GB cells, GFAP sustained in glial cells	Yes	Pro: single-cell transcriptome analysis	[3, 13–18]

Radial glial cell	Similar to GB cells: GFAP+ SOX2+ NESTIN+	Limited, similar to GB cells *in vitro*	Multipotent differentiation similar to GB cells, GFAP sustained in glial cells	Yes	Pro: single-cell NGS Against: it is not definite if these cells exist in adults brain	[3–7, 19–27]

Classical NSC	Different from GB cells: SOX2+ NESTIN+	Not limited	Multipotent GFAP gain in glial cells	Yes	Against: in secondary GB IDH1R132H triggers apoptosis	[8, 11, 18, 28–36]

Astrocyte	Different from GB cells: GFAP+	Limited	Mature	Yes	Pro: ErbB expression changed astrocytes into radial glia like cells	[37–39]
